# Functional and Structural Consequence of Rare Exonic Single Nucleotide Polymorphisms: One Story, Two Tales

**DOI:** 10.1093/gbe/evv191

**Published:** 2015-10-09

**Authors:** Wanjun Gu, Christopher I. Gurguis, Jin J. Zhou, Yihua Zhu, Eun-A. Ko, Jae-Hong Ko, Ting Wang, Tong Zhou

**Affiliations:** ^1^Research Center for Learning Sciences, Southeast University, Nanjing, Jiangsu, China; ^2^Department of Medicine, The University of Arizona; ^3^Department of Epidemiology and Biostatistics, The University of Arizona; ^4^School of Biological Science and Medical Engineering, Southeast University, Nanjing, Jiangsu, China; ^5^College of Information Science and Technology, Nanjing Agricultural University, Nanjing, Jiangsu, China; ^6^Department of Pharmacology, The University of Nevada School of Medicine, Reno; ^7^Department of Physiology, College of Medicine, Chung-Ang University, Seoul, South Korea

**Keywords:** single nucleotide polymorphisms, purifying selection, positive selection, ancestral allele, translational selection, RNA structure

## Abstract

Genetic variation arising from single nucleotide polymorphisms (SNPs) is ubiquitously found among human populations. While disease-causing variants are known in some cases, identifying functional or causative variants for most human diseases remains a challenging task. Rare SNPs, rather than common ones, are thought to be more important in the pathology of most human diseases. We propose that rare SNPs should be divided into two categories dependent on whether the minor alleles are derived or ancestral. Derived alleles are less likely to have been purified by evolutionary processes and may be more likely to induce deleterious effects. We therefore hypothesized that the rare SNPs with derived minor alleles would be more important for human diseases and predicted that these variants would have larger functional or structural consequences relative to the rare variants for which the minor alleles are ancestral. We systematically investigated the consequences of the exonic SNPs on protein function, mRNA structure, and translation. We found that the functional and structural consequences are more significant for the rare exonic variants for which the minor alleles are derived. However, this pattern is reversed when the minor alleles are ancestral. Thus, the rare exonic SNPs with derived minor alleles are more likely to be deleterious. Age estimation of rare SNPs confirms that these potentially deleterious SNPs are recently evolved in the human population. These results have important implications for understanding the function of genetic variations in human exonic regions and for prioritizing functional SNPs in genome-wide association studies of human diseases.

## Introduction

What genetic variants are most functionally relevant for human diseases? With rapid advances in next-generation DNA sequencing technologies, whole exome, or genome sequencing have been widely applied in genetic association studies to identify the causative genetic variations of human diseases or traits (Goldstein et al. 2013; Soon et al. 2013). A majority of currently known disease-associated variants are common single nucleotide polymorphisms (SNPs), yet these SNPs can only explain a small proportion of genetic variance in diseases (Manolio et al. 2009). One possibility is that rare SNPs, rather than common ones, are more important in the pathology of most human diseases (Cirulli and Goldstein 2010; Gibson 2010, 2012).

Several lines of evidences support this “common disease-rare variants” hypothesis (Iyengar and Elston 2007). First, many studies have related rare genetic variants to complex human diseases, such as type 2 diabetes, hypertriglyceridemia, sick sinus syndrome (McClellan et al. 2007; Nejentsev et al. 2009; Johansen et al. 2010; Holm et al. 2011; Rivas et al. 2011; Need et al. 2012; Lohmueller et al. 2013). Second, disease-causing variants should be selectively unfavorable (i.e., should decrease fitness). Therefore, purifying selection may be common in the evolution of SNPs, preventing deleterious alleles from drifting to a higher frequency in the population (Bulmer 1971). Third, theoretical population genetics suggests that recent explosion of human population size may allow the accumulation of rare alleles with deleterious effects (Lynch 2010; Simons et al. 2014). Finally, accumulating human polymorphism data from 1000 Genomes Project (1000 Genomes Project Consortium 2012), Personal NHLBI Exome Sequencing Project (Tennessen et al. 2012; Fu et al. 2013), and International HapMap Project (International HapMap 3 Consortium 2010) exemplify that the majority of human SNPs are rare. Rare SNPs are enriched in genomic regions that are evolutionarily conserved (Zhu et al. 2011; 1000 Genomes Project Consortium 2012) or functionally important (Wray 2007; Montgomery et al. 2011; Maurano et al. 2012). Therefore, rare SNPs should be considered as a high priority when identifying causative genetic variants in genetic association studies and medical genome sequencing (Pabinger et al. 2014).

In general, SNPs with the minor allele frequency (MAF) less than 1% are defined to be rare variants (Frazer et al. 2009). Among rare variants, some are evolutionarily conserved, suggesting that if they are deleterious, they have small functional effects or are maintained in the genome by various mechanisms (e.g., compensatory or epistatic effects or antagonistic pleiotropy [Davis et al. 2009]). These SNPs may be less strongly associated with phenotypic changes. Newly arisen variants in the genome, however, have had less time to be purged by selection or other evolutionary processes, and may harbor more deleterious effects. Therefore, we hypothesized that rare SNPs that have arisen recently should be given priority when determining what genetic variants are functionally relevant for human diseases. We predicted that 1) MAF would be negatively associated with deleteriousness only for derived alleles, and 2) the derived allele frequency (DAF) would better predict phenotypic effects of SNPs.

The potential deleteriousness of a variant is related to the strength of its functional effects (Fisher 1930). Previous efforts have primarily focused on nonsynonymous variants occurring in protein coding regions (International HapMap 3 Consortium 2010; Zhu et al. 2011; 1000 Genomes Project Consortium 2012; Tennessen et al. 2012; Fu et al. 2013). However, little is known about the functional consequences of synonymous SNPs or SNPs outside coding regions. Several previous studies have used conservation scores, which are derived from evolutionary comparisons, in estimating the functional importance of such SNPs (Chorley et al. 2008; Goode et al. 2010; Montgomery et al. 2011; Maurano et al. 2012; Kircher et al. 2014) because it is difficult to quantify the functional consequences of noncoding or synonymous SNPs. Recent high-throughput RNA structure profiling data and bioinformatics analyses (Li et al. 2012; Mortimer et al. 2014) have revealed important regulatory roles performed by RNA structures in different mRNA regions, such as mRNA translation initiation regions (Gu et al. 2010), microRNA target site regions (Gu et al. 2012), 5′-untranslated regions (5′-UTRs) near the cap site (Gu et al. 2014b), and RNA splicing regions (Li et al. 2013). Single nucleotide variations, including several synonymous variants, have been proposed to cause diseases by altering mRNA structure (Sauna and Kimchi-Sarfaty 2011; Salari et al. 2013). Hence, a systematic analysis of structural effects caused by exonic variants, including synonymous SNPs, nonsynonymous SNPs, SNPs in 5′-UTRs, and SNPs in 3′-UTRs will help provide insights into the functional and structural effects of rare exonic SNPs in the human genome.

Here, we performed a genome-wide analysis of functional and structural changes caused by all exonic SNPs in the human genome. We calculated changes to mRNA structure made by each exonic SNP in silico. We also calculated the effects on mRNA translation for synonymous SNPs, and alterations on protein function for nonsynonymous SNPs. For the SNPs for which the minor alleles is derived, functional changes made by rare exonic variants are stronger than those made by common variants. This pattern suggests purifying selection, rather than positive or balancing selection, as a major driver in shaping exonic variation in human populations (Zhu et al. 2011). Notably, this pattern is reversed when we focus only on those variants for which the minor alleles are ancestral. Exonic variants with DAF larger than 99% have the least functional consequences. Therefore, rare variants for which the minor alleles are ancestral are less likely to be deleterious (Gorlova et al. 2012). We propose that these variants can be excluded when prioritizing causative variants in genetic association studies. In addition, we observed a significant enrichment of rare variants, which have the biggest functional and structural effects, in exonic regions of human genome. Age distribution analysis of these rare variants confirmed a recent explosion of deleterious SNPs in the human population (1000 Genomes Project Consortium 2012; Fu et al. 2013).

## Materials and Methods

### SNP and Random Mutation Data

We obtained exonic SNPs within protein-coding genes from dbSNP (May 21, 2014, release; NCBI dbSNP Human Build 141) (Sherry et al. 2001). The ancestral state of each SNP was obtained from Ensembl (Flicek et al. 2014), which was based on a six-way primate genome-wide alignment (Paten et al. 2008). Only the SNPs with annotated ancestral allele and global MAF were included. We excluded the SNPs with more than two alleles. The SNPs without unique gene annotation were also excluded. We matched each SNP to the corresponding transcripts defined by Ensembl (Flicek et al. 2014). Only the longest transcript for each SNP was saved for further analysis. In total, we collected 720,480 exonic SNPs, including 46,157 SNPs located in 5′-UTR, 261,697 SNPs located in 3′-UTR, 240,813 nonsynonymous SNPs, and 171,813 synonymous SNPs. We also randomly put 100,000 random point mutations into the transcripts, which guaranteed the same transition/transversion ratio in 5′-UTR, 3′-UTR, and coding region. In total, we obtained 53,307 and 46,693 random point mutations in coding and UTRs, respectively.

### PhyloP, GERP, CADD, and SIFT Scores

We downloaded CADD (Kircher et al. 2014) score with annotations for all possible human (version hg19) SNPs from CADD website (http://cadd.gs.washington.edu/download, last accessed October 12, 2015), and parsed PhyloP (Siepel et al. 2006), GERP (Cooper et al. 2010), CADD (Kircher et al. 2014), and SIFT (Ng and Henikoff 2003) scores for each exonic human SNP.

### Amino Acid Chemical Distance, Hydrophobicity Index, and BLOSUM62 Score

We used the amino acid chemical distance, change in amino acid hydrophobicity index (|*ΔH*|), and BLOSUM62 score to evaluate the potential functional effect caused by nonsynonymous SNPs. The definition of amino acid chemical distance was obtained from a previous study (Grantham 1974). The table of amino acid hydrophobicity index was obtained from Argos et al’s study (Argos et al. 1982). BLOSUM62 is a weighting matrix for amino acid substitution (Henikoff S and Henikoff JG 1992). A BLOSUM62 score of zero indicates that the frequency of the given amino acid substitution does not differ from that expected by chance, while a positive score indicates that the substitution occurs more frequently than by chance, while negative score indicates that the substitution is found less often than by chance.

### Evolutionary Rate

Nonsynonymous substitutions rate (*dN*) and synonymous substitutions rate (*dS*) of each human transcript were obtained from BioMart of Ensembl (Flicek et al. 2014). Both mouse and chimp orthologs were used in this study.

### Prediction of mRNA Secondary Structure

Two software, remuRNA (Salari et al. 2013) and RNAsnp (Sabarinathan et al. 2013), were used to predict the effect of single-point substitution on local mRNA secondary structure. remuRNA computes the structural relative entropy between the Boltzmann ensembles of the native and a mutant structure (Salari et al. 2013). As suggested by Salari et al. (2013), we used the local version of remuRNA with a window size of 150 nt to compute the mRNA local structural entropy caused by SNPs or random mutations, using the default settings. RNAsnp focuses on the local regions of maximal structural change between the native and a mutant mRNA. Structural distance between wild-type and mutant sequences was calculated from base pairing probability matrices (Sabarinathan et al. 2013). We applied “Mode 2” in RNAsnp with default settings to compute the structural distance caused by SNPs or random mutations.

### Codon Optimality

We used the method presented in our previous study (Zhou et al. 2009) to compute codon optimality. We first obtained the expression data for human genes from Su et al. (2014). We measured expression level as the geometric mean of expression among different tissues. Next, we compared the codon usage pattern between the gene groups showing the lowest 20% and highest 20% expression level. Codon optimality was defined as the odds ratio of codon usage between highly and lowly expressed groups, calculated separately for each codon (supplementary table S1, Supplementary Material online).

### Translation Initiation Sites and Splicing Sites

We defined translation initiation sites (TISs) as the 5′-UTR regions of 50 bp upstream of the start codon. Splicing sites (SSs) were defined as exonic regions of 3 bp from exon–intron boundary.

### Gene Categories

First, we stratified human genes into subgroups according to their functional roles as essential or nonessential genes. The definition of functionally essential genes was obtained from Liao and Zhang (2008). Next, human genes were categorized according to their involvement in Mendelian diseases as Mendelian or non-Mendelian genes. The genes involved in human Mendelian genetic disorders were obtained from OMIM.org (Amberger et al. 2015).

### SNP Age

We downloaded the age information for protein-coding SNPs from the NHLBI Exome Sequencing Project (Tennessen et al. 2012; Fu et al. 2013), which was estimated based upon the demographic models with recent accelerated population growth. The SNP age data are available for both European American and African American. In this study, we mainly focused on the age data in European American.

### Statistical Analysis

All the statistical analyses were performed within the R platform. Spearman’s rank correlation test, *t*-test, and Wilcoxon rank sum test were conducted using “cor.test,” “t.test,” and “wilcox.test” functions, respectively. Cumulative distributions were computed by “ecdf” function, which is a step function with jumps *i*/*n* at observation values. Here, *i* is the number of tied observations whereas *n* is the total number of observations.

## Results

### Selective Constraint Is Not Necessarily a Function of MAF

Rare SNPs are thought to be more deleterious and more likely to be associated with human genetic disorders (Kryukov et al. 2007; Zhu et al. 2011; Subramanian 2012). However, these SNPs can be stratified into two groups depending on whether the minor allele is derived or ancestral. Because there is a potential difference in the history of selection on these two categories of SNPs (Zhu et al. 2011), we examined each category separately.

**Fig. 1.—:**
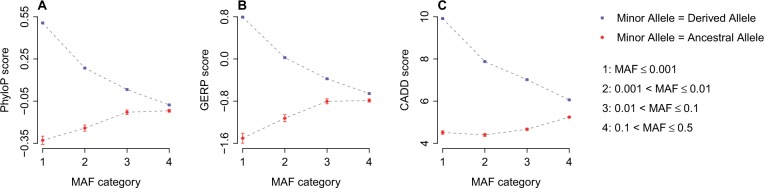
Correlation between MAF of exonic SNPs and corresponding conservation/pathogenicity score. (*A*) Relationship between MAF and PhyloP score; (*B*) Relationship between MAF and GERP score; (*C*) Relationship between MAF and CADD score. Each point represents the mean of the corresponding category. Error bars indicate the standard error of the mean.

First, PhyloP (Goode et al. 2010) and GERP (Cooper et al. 2005) scores were used to evaluate the conservation of and selective constraints on each variant site, respectively. Larger PhyloP scores suggest higher degree of conservation, while larger GERP scores suggest stronger evolutionary constraints. Consistent with previous findings (Cooper et al. 2010; Zhu et al. 2011), MAF is negatively correlated (*P* < 10^−^^10^ by Spearman’s rank correlation test) with PhyloP and GERP scores for exonic SNPs for which the minor alleles are derived (fig. 1*A* and *B*). However, MAF was positively correlated (Spearman’s rank correlation test: *P* < 10^−^^10^) with PhyloP and GERP scores for exonic SNPs for which the minor alleles are ancestral (fig. 1*A* and *B*).

Next, we measured the pathogenicity of exonic SNPs using the CADD (Kircher et al. 2014) score. A higher CADD score implies a more deleterious effect caused by the SNP. The relationship between MAF and CADD score mirrors the pattern we found for PhyloP and GERP scores (fig. 1*C*). MAF and CADD scores are negatively correlated (Spearman’s rank correlation test: *P* < 10^−^^10^) for SNPs for which the minor alleles are derived, but positively correlated (Spearman’s rank correlation test: *P* < 10^−^^10^) for SNPs for which the minor alleles are ancestral. The positive correlation between MAF and CADD score of SNPs for which the minor alleles are ancestral may be interpreted by purifying selection: only the alleles with the weakest deleterious effect can drift to a high frequency (a low ancestral allele frequency) in human populations. Also, some rare SNPs with ancestral minor alleles may experience positive selection in human evolution. Therefore, rare SNPs with ancestral minor alleles tend to have the weakest pathogenicity and the lowest CADD score (fig. 1*C*), which can be reflected by the positive correlation between MAF and CADD score.

Taken together, these results provide evidence that rare exonic SNPs should be categorized into two distinct groups—those with derived minor alleles and those with ancestral minor alleles. Selective constraint or pathogenicity is not necessarily a function of MAF. Instead, they are a function of DAF (supplementary fig. S1, Supplementary Material online). DAF may, by itself, serve as a predictor of functional consequence of exonic SNPs (Kryukov et al. 2007). Although we observed an enormous excess of rare exonic SNPs (MAF < 0.01) at the both extremes of the DAF (supplementary fig. S2, Supplementary Material online), the minor alleles of most rare SNPs (99.1%) are derived. In this study, in total, there are 577,956 exonic SNPs with DAF < 0.01, but only 5,500 exonic SNPs with DAF > 0.99. From this point on, we only focus on DAF when examining the functional and structural consequences of rare exonic SNPs.

### DAF Is Modulated by Functional Effects of Nonsynonymous SNPs

Nonsynonymous sites in coding sequences are thought to be under very strong purifying selection. In this study, 33.5% of the exonic SNPs are nonsynonymous. Here, we assessed the relationship between DAF and the functional properties of nonsynonymous SNPs. Nonsynonymous SNPs were divided into eight bins according to their DAF. The SNP-induced functional alteration were measured by amino acid chemical distance (Grantham 1974), change in amino acid hydrophobicity index (|*ΔH*|) (Argos et al. 1982), BLOSUM62 score (Henikoff S and Henikoff JG 1992), and SIFT score (Ng and Henikoff 2003). Random point mutations at nonsynonymous sites were used as a background.

**Fig. 2.—:**
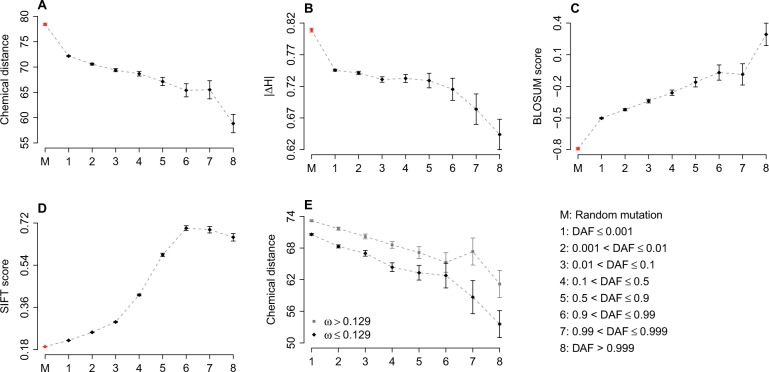
Functional effects caused by nonsynonymous SNPs as a function of DAF. (*A*) Amino acid chemical distance caused by nonsynonymous SNPs as a function of DAF; (*B*) Change in amino acid hydrophobicity index (|*ΔH*|) as a function of DAF; (*C*) BLOSUM62 scores as a function of DAF; (*D*) SIFT scores as a function of DAF; (*E*) Comparison of the functional effect caused by nonsynonymous SNPs between the genes undergoing stronger and weaker purifying selection. The median of ω (calculated using mouse orthologs) is used as the cutoff. Each point represents the mean of the corresponding category. Error bars indicate the standard error of the mean.

Chemical distance and |*ΔH*| induced by nonsynonymous SNPs decreased monotonically with increasing DAF (Spearman’s rank correlation test: *P* < 10^−^^10^ for chemical distance and *P* = 0.002 for |*ΔH*|) (fig. 2*A* and *B*). The physicochemical alterations caused by random nonsynonymous mutation are significantly higher (*t*-test: *P* < 10^−^^10^) than that of nonsynonymous SNPs (fig. 2*A* and *B*).

BLOSUM62 scores measures the likelihood of substitution between two amino acids. Larger BLOSUM62 scores indicate a higher likelihood of substitution at a specific site. SIFT scores predicts whether an amino acid substitution affects protein function (Ng and Henikoff 2003). Lower SIFT scores imply more deleterious effect caused by nonsynonymous substitution. Figure 2*C* and *D* reveal that BLOSUM62 and SIFT scores are positively correlated with DAF (Spearman’s rank correlation test: *P* < 10^−^^10^). The BLOSUM62 and SIFT scores of random nonsynonymous mutations are significantly lower (*t*-test: *P* < 10^−^^10^) than that of nonsynonymous SNPs in our study (fig. 2*C* and *D*).

We used the *ω* value, ratio of nonsynonymous substitutions rate (*dN*) to synonymous substitutions rate (*dS*), to evaluate the selective pressure acting on coding sequences. When *ω* < 1, this is indicative of purifying selection (Kryazhimskiy and Plotkin 2008). We first focused on the *ω* data generated between human and mouse. Interestingly, we found that the functional effect caused by nonsynonymous SNPs of genes undergoing stronger purifying selection is significantly weaker (*t*-test: *P* < 10^−^^10^) than that of genes under weaker purifying selection (fig. 2*E* and supplementary fig. S3, Supplementary Material online). A similar pattern was observed when human transcripts were compared with chimp orthologs (supplementary fig. S4, Supplementary Material online). All together, these results suggest that purifying selection prevents deleterious derived alleles at nonsynonymous sites from drifting to a higher frequency in human populations.

### DAF Is a Function of Alterations to mRNA Structure Caused by Exonic SNPs

**Fig. 3.—:**
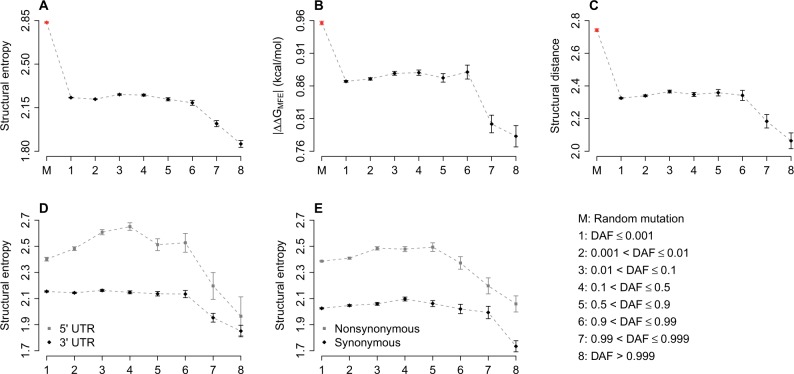
The mRNA structural effect caused by exonic SNPs as a function of DAF. (*A*) Structural entropy caused by exonic SNPs as a function of DAF; (*B*) MFE gap between two alleles (|*ΔΔG_MFE_*|) as a function of DAF; (*C*) Structural distance between two alleles as a function of DAF; (*D*) Comparison of the structural entropy between SNPs located in 5′- and 3′-UTR; (*E*) Comparison of the structural entropy between nonsynonymous and synonymous SNPs. Each point represents the mean of the corresponding category. Error bars indicate the standard error of the mean.

Selection against mutations that significantly change mRNA structure is widespread (Chamary and Hurst 2005; Kudla et al. 2009; Gu et al. 2010, 2012, 2014a, 2014b; Zhou and Wilke 2011). Here, we examined the relationship between DAF of exonic SNPs and corresponding alteration in mRNA secondary structure. Two independent programs, remuRNA (Salari et al. 2013) and RNAsnp (Sabarinathan et al. 2013), were used to predict the effect of single-point mutation on local mRNA secondary structure. remuRNA computes the structural entropy in Boltzmann ensembles between wild-type and mutant structure (Salari et al. 2013). Larger structural entropy indicates a more substantial alteration in mRNA secondary structure. RNAsnp focuses on the local regions of maximal structural change caused by mutation (Sabarinathan et al. 2013). Structural distance between wild-type and mutant sequences was calculated from base pairing probability matrices (Sabarinathan et al. 2013). Larger structural distance indicates higher dissimilarity in mRNA secondary structure. Figure 3*A* indicates that the structural entropy of random point mutations is significantly higher (*t*-test: *P* < 10^−^^10^) than that of exonic SNPs. Together, the structural entropy of the SNPs in bins 7 (0.99 < DAF ≤ 0.999) and 8 (DAF > 0.999) is significantly lower (*t*-test: *P* < 10^−^^10^) than the structural entropy of the other bins (bin 1–6).

Similar patterns were observed when we looked into the mRNA structure of minimum free energy (MFE) (fig. 3*B*). The folding energy gap of MFE structure (|*ΔΔG_MFE_*|) in bin 7 and 8 is much lower (*t*-test: *P* < 10^−^^6^) than that in the other bins (fig. 3*B*). In addition, as shown in figure 3*C*, the structural distance predicted by RNAsnp largely mirrors the pattern in figure 3*A* and *B.* These results demonstrate that DAF is closely related to the structural effects of rare exonic SNPS, and also suggest strong selection against mutations that significantly change mRNA structure. Derived alleles with less structural effect are more likely to eventually become fixed in human population.

A comparison of SNPs located in 5′- and 3′-UTR revealed that the structural effect of the SNPs in 5′-UTR is significantly higher (*t*-test: *P* < 10^−^^10^) than those in 3′-UTR (fig. 3*D*). This finding suggests that the 3′-UTR undergoes stronger purifying selection against mRNA structural alterations compared with the 5′-UTR. Furthermore, the structural effect caused by nonsynonymous SNPs is significantly higher (*t*-test: *P* < 10^−^^10^) than the effect of synonymous SNPs (fig. 3*E*). Thus, synonymous sites seem to be subject to stronger selection in keeping RNA secondary structures compared with nonsynonymous sites in coding sequences.

### Selection on Translational Efficiency in Synonymous SNPs

**Fig. 4.—:**
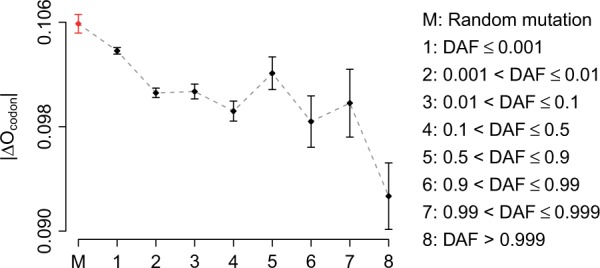
Change in codon optimality (|*ΔO*_codon_|) as a function of DAF. Each point represents the mean of the corresponding category. Error bars indicate the standard error of the mean.

In this study, 23.8% of the exonic SNPs are synonymous. Selection on synonymous sites for translational efficiency has been observed among a diversity of prokaryotes and eukaryotes, including for humans (Drummond and Wilke 2008; Zhou et al. 2009; Lee et al. 2010). In general, translational optimal codons are the ones that are efficiently and rapidly translated because their cognate tRNAs are highly abundant (Zhou et al. 2010). Optimal codons may confer a selective advantage to genes for which high translational speed and accuracy is required. However, there is also evidence that nonoptimal codons are selected at specific coding sites to facilitate cotranslational protein folding (Thanaraj and Argos 1996; Komar et al. 1999; Zhang et al. 2009). Here, we investigated the relationship between DAF and selection on translational efficiency in synonymous variants. We followed the method presented in one previous study (Zhou et al. 2009) to define codon optimality (see Materials and Methods for details). Change in codon optimality (|*ΔO*_codon_|) was calculated for each synonymous SNPs. A significant negative correlation (Spearman’s rank correlation test: *P* < 10^−^^10^) was found between DAF and |*ΔO*_codon_| (fig. 4). Also, |*ΔO*_codon_| of random synonymous mutations is significantly higher (*t*-test: *P* < 10^−^^10^) than that of synonymous SNPs (fig. 4). These results suggest that purifying selection on synonymous sites, which may substantially affect translational efficiency or cotranslational folding, prevents those derived alleles from drifting to a higher frequency in human populations.

### Regional Difference in DAF

All of the above observations indicate that DAF is modulated by the functional and structural consequences of exonic SNPs. For this reason, DAF may serve as a predictor of functional significance of exonic SNPs. Therefore, comparison of DAF among different mRNA sites may help us understand the patterns of selection acting on exonic SNPs.

**Fig. 5.—:**
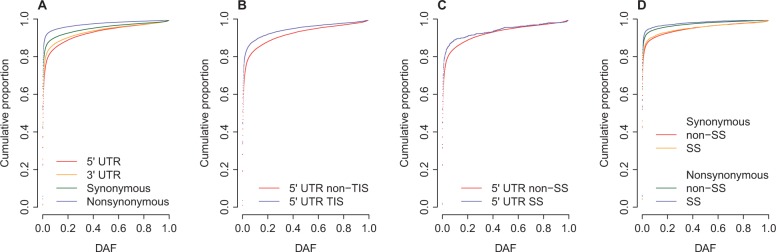
Cumulative distribution of DAF. (*A*) Comparison of DAF between SNPs located in 5′-UTR, 3′-UTR, and coding regions; (*B*) Comparison of DAF between 5′-UTR SNPs within and outside of TIS; (*C*) Comparison of DAF between 5′-UTR SNPs located within and outside of splice site (SS); (*D*) Comparison of DAF between coding SNPs located within and outside of SS.

We found that the DAF of nonsynonymous SNPs is significantly lower (Wilcoxon rank sum test: *P* < 10^−^^10^) than the DAF of synonymous ones (fig. 5*A*). Also, the DAF of SNPs in 3′-UTR is significantly lower (Wilcoxon rank sum test: *P* < 10^−^^10^) than the DAF of SNPs in 5′-UTR (fig. 5*A*). Together, these results suggest that the genomic positions of nonsynonymous SNPs are under the least evolutionarily labile while the positions of SNPs in 5′-UTR are undergoing the weakest selection.

Although 5′-UTR may have been under weaker purifying selection, the 5′-UTR is important for the regulation of translation in eukaryotes. For example, the TISs, located in the 5′-UTR immediately upstream of the start codon, are under strong selection to facilitate translation initiation (Dvir et al. 2013; Gu et al. 2014a). Here, we define TISs as the regions of 50 bp upstream of the start codon. Figure 5*B* indicates that the DAF of the SNPs within TIS (*n* = 8,565) is significantly lower (Wilcoxon rank sum test: *P* < 10^−^^10^) than the DAF of non-TIS variants, which is consistent with the action of purifying selection.

Splice sites (SSs) in mRNA are also thought to be under strong purifying selection (Orban and Olah 2001; Parmley et al. 2006; Lawrie et al. 2013). Here, SSs are defined as exonic regions of 3 bp from exon–intron boundary. We found that, in 5′-UTR, the DAF of the SNPs within SS (*n* = 744) is significantly lower (Wilcoxon rank sum test: *P* < 10^−^^10^) than the DAF of non-SS variants (fig. 5*C*). Also, in coding region, the DAF of the SNPs within SS (*n* = 4,480 for synonymous SNPs and *n* = 6,418 for nonsynonymous SNPs) is significantly lower (Wilcoxon rank sum test: *P* = 1.8 × 10^−5^ for synonymous SNPs and *P* < 10^−^^10^ for nonsynonymous SNPs) than the DAF of non-SS variants (fig. 5*D*). However, we did not find significant difference in DAF between SS and non-SS SNPs in 3′-UTR (Wilcoxon rank sum test: *P* = 0.681), which may be due to the limited number of SS variants in 3′-UTR (*n* = 107).

### Difference in DAF between Gene Categories

We investigated the difference in DAF among different gene categories. First, we categorized the SNPs according to the functional essentiality of their host gene. We found that the DAF of SNPs in essential genes (*n* = 5,504) is significantly lower (Wilcoxon rank sum test: *P* = 5.4 × 10^−^^3^) than the DAF of SNPs in non-essential genes (supplementary fig. S5*A*, Supplementary Material online). Next, we checked the difference between Mendelian and non-Mendelian genes. We found that the DAF of SNPs in Mendelian genes (*n* = 522,696) is significantly lower (Wilcoxon rank sum test: *P* < 10^−^^10^) than that in non-Mendelian genes (supplementary fig. S5*B*, Supplementary Material online).

### DAF and SNP Age

SNP age was previously reported to be negatively correlated with SNP-induced deleterious effect (Fu et al. 2013). We collected the age information for protein-coding SNPs from the NHLBI Exome Sequencing Project (Tennessen et al. 2012; Fu et al. 2013). The SNP age data are available for both European American and African American. Here, we focused on the age data in European American first.

**Fig. 6.—:**
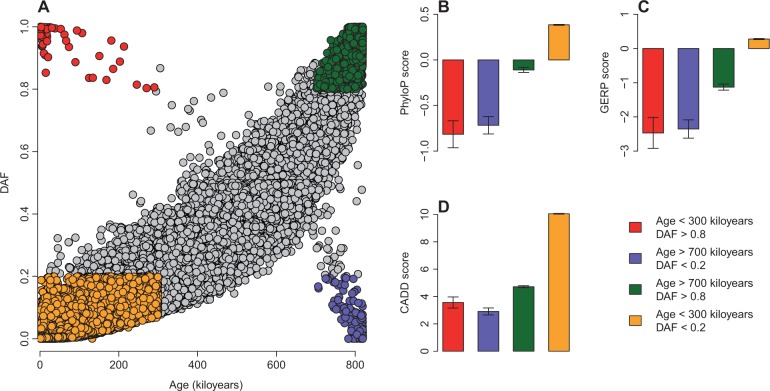
Relationship between SNP age and DAF. (*A*) DAF increases with SNP age. Outliers are aggregated at the top left (red points with age < 300 kiloyears and DAF > 0.8) and bottom right (blue points with age > 700 kiloyears and DAF < 0.2) corners. SNPs located in the top right (green points with age > 700 kiloyears and DAF > 0.8) and bottom left corners (orange points with age < 300 kiloyears and DAF < 0.2) are highlighted. (*B*) Comparison of PhyloP score among SNPs located in the four corners in panel A. (*C*) Comparison of GERP score among SNPs located in the four corners in panel A. (*D*) Comparison of CADD score among SNPs located in the four corners in panel A.

Not surprisingly, we found a strong positive correlation (Spearman’s rank correlation test: *P* < 10^−^^10^) between SNP age and DAF (fig. 6*A*). A negative relationship (Spearman’s rank correlation test: *P* < 10^−^^10^) between age and PhyloP, GERP, and CADD scores was also observed (supplementary fig. S6, Supplementary Material online). However, figure 6*A* indicates that there are several outliers aggregated at the top left (age < 300 kiloyears and DAF > 0.8) and bottom right (age > 700 kiloyears and DAF < 0.2) corners of the figure. The SNPs at the top left are those in which derived allele became major allele quickly (“quick-running” SNP). The SNPs at the bottom right corner are old variants with relatively low DAF (“slow-running” SNP). Further investigation reveals that the deleterious effect caused by both quick-running and slow-running SNPs is significantly weaker (*t*-test: *P* < 10^−^^2^) than that of the SNPs aggregated at the top right (age > 700 kiloyears and DAF > 0.8) and bottom left (age < 300 kiloyears and DAF < 0.2) corners (supplementary fig. S7, Supplementary Material online and fig. 6*B*–*D*), which suggests that, even with similar age, the SNPs at the two DAF extremes differ in the strength of purifying selection. Pathway enrichment analysis was conducted for the genes of quick-running SNPs based on KEGG database (Kanehisa et al. 2014). Only the genes of SNPs with DAF > 0.8 were used as the background of enrichment study. We observed that “Osteoclast differentiation,” “Bladder cancer,” “Butanoate metabolism,” and “Olfactory transduction” are among the top pathways enriched in the genes of quick-running SNPs (supplementary fig. S8*A*, Supplementary Material online). We also conducted pathway analysis for the genes of slow-running SNPs. Here, the genes of SNPs with DAF < 0.2 were used as the background of enrichment study. Interestingly, Olfactory transduction is the most significant pathway enriched in the genes of slow-running SNPs (supplementary fig. S8*B*), Supplementary Material online. The above findings can be largely reproduced when the age data in African American are used (supplementary figs. S9–S12, Supplementary Material online).

## Discussion

All our analyses demonstrated a robust and consistent pattern of functional and structural changes with regard to the frequency of derived allele (figs. 2–4). The changes on protein structure and/or function, RNA local structure, and translation efficiency are larger for SNPs at lower DAF in the human population. Compared to artificially generated, randomized mutations in the human genome, naturally observed human SNPs, even those SNPs with very small DAFs, have smaller functional/structural changes (figs. 2–4). Several previous studies have shown that rare SNPs with derived minor alleles are more likely to be located in evolutionary conserved region (Zhu et al. 2011; Tennessen et al. 2012; Fu et al. 2013, 2014). In particular, Zhu et al. (2011) sequenced whole genomes of 29 individuals of European origin and assessed allele frequencies. Using SNP information in this small-size population, they found that genomic positions with low-DAF SNPs are more conserved than positions corresponding to high-DAF SNPs and rare SNPs with derived minor alleles are more likely to be located in functional genomic regions (Zhu et al. 2011). Furthermore, Fu et al*.* (2014) analyzed human coding SNPs from 6,515 individuals of European and African origin and identified putative deleterious SNPs in this population based on PhyloP conservation score (Fu et al. 2014). They also showed that derived minor alleles were more deleterious than ancestral minor alleles. In comparison to these previous studies, we performed a genome-wide and systematic analysis of the functional and structural consequences of all known human exonic SNPs. Other than coding nonsynonymous SNPs, we also analyzed coding synonymous SNPs and noncoding SNPs in 5′- and 3′-UTRs. In addition to evolutionary conservation, we focused on the consequences of human exonic SNPs at three biological levels. First, nonsynonymous SNPs in protein coding regions change encoded amino acids, which in turn will have effects on the structure and function of the final protein products (Bromberg and Rost 2007). We computed several measures for each nonsynonymous SNP in estimating its effect on protein structure and function, including amino acid chemical distance (Grantham 1974), change in amino acid hydrophobicity index (|*ΔH*|) (Argos et al. 1982), BLOSUM62 score (Henikoff S and Henikoff JG 1992), and SIFT score (Ng and Henikoff 2003). Second, RNA structures are very important in several biological processes, such as RNA splicing (Parmley et al. 2006), RNA translation initiation (Gu et al. 2010), and microRNA-mediated gene regulation (Gu et al. 2014b). Human exonic mutations can affect those biological processes by altering local mRNA structures. We computed the change of RNA structure for each exonic SNP with several different measures (Sabarinathan et al. 2013; Salari et al. 2013) and estimated its effect on local RNA structure through these calculations. Third, synonymous SNPs in the protein coding region can regulate translational process of messenger RNA (Gingold and Pilpel 2011). Biased usages of synonymous codons are related to translation efficiency and accuracy, and thus cotranslational protein folding process (Hershberg and Petrov 2008). We investigated the effect on mRNA translation made by synonymous SNPs in the human genome by calculating the change in codon optimality (|*ΔO*_codon_|) (Zhou et al. 2009). Our results showed that derived, young alleles had higher disruptive effects than ancestral alleles at all three biological levels, which explained why derived minor alleles are the most deleterious. This observation complemented previous findings (Zhu et al. 2011; Gorlova et al. 2012; Fu et al. 2014), which taken together suggested that derived rare alleles in human genomes had higher probability in causing human diseases than ancestral rare alleles.

Minor allele can be separated into two groups according to ancestral state. Our results show that these SNPs have completely different functional consequences on protein structure/function, RNA translation, and mRNA secondary structure (figs. 1–4). Variants with the DAF less than 1% have the biggest functional changes, whereas variants with DAF larger than 99% have the smallest functional consequences. Furthermore, we observed more than 100-fold enrichment of rare variants with DAF less than 0.1% in the human genome, but only 2- to 10-fold enrichment for variants with DAF larger than 99.9%. Genetic variants in the human population are enriched with rare variants, especially those with highest functional changes. These observations suggest that the causative variants for most human complex diseases may be rare derived variants with large functional effects, such as those with DAF less than 0.1%. This supports the common disease-rare variants hypothesis. Human SNPs with DAF larger than 99.9% are less likely to be deleterious and therefore less likely to be causally related to human diseases. When prioritizing variants in genetic association studies or whole exome/genome sequencing, we should consider whether a variant is derived in addition to its frequency.

Many studies have observed that synonymous codons are selected for loose or tight RNA structure to facilitate RNA splicing, RNA translation initiation, and microRNA binding (Chamary and Hurst 2005; Chamary et al. 2006; Parmley et al. 2006; Gu et al. 2010, 2012, 2014a, 2014b; Mortimer et al. 2014). When filtering functional synonymous SNPs that may be related to human diseases, secondary structure alteration is an important feature to estimate its pathogenicity (Buske et al. 2013). RNA secondary structure change caused by nonsynonymous SNPs is rarely considered in functional annotation of nonsynonymous SNPs (Frousios et al. 2013). Recent studies have implicated the important role of RNA structure in gene regulation (Mortimer et al. 2014; Yang et al. 2014; Wang et al. 2015), and nonsynonymous mutations in human exonic region should be selected for functional RNA structures (Gu et al. 2014a). Our results show that both nonsynonymous and synonymous SNPs altered local RNA structure, and the structural entropy difference caused by nonsynonymous SNPs is higher than synonymous SNPs (fig. 3*E*). Hence, we propose that nonsynonymous SNPs could also cause abnormal gene expression or function by altering RNA secondary structure, which should be considered in functional annotation of nonsynonymous SNPs.

In addition to synonymous and nonsynonymous SNPs in protein coding region, human SNPs in 5′-UTR and 3′-UTR have substantial effects on local RNA secondary structure. Previous studies have found important biological roles performed by RNA structure in 5′-UTR and 3′-UTR. RNA structure near the cap site is crucial for microRNA-mediated gene regulation in humans (Gu et al. 2014b). RNA secondary structure upstream of the start codon is also important in efficient translation initiation(Goodman et al. 2013). microRNA target sites are located in 3′-UTR of most human genes, implicating these sites as an important target of epigenetic regulation (Bartel 2009). Many studies have confirmed that secondary structure of microRNA targets and their flank region is related to microRNA target accessibility and microRNA binding. In our results, we observed that structural effects caused by SNPs in 5′- or 3′-UTR are similar to those caused by SNPs in protein coding region (fig. 3*D*). This finding hinted that altering RNA structure is an important mechanism by which exonic SNPs can cause aberrant gene expression and human disease.

Interestingly, human nonsynonymous SNPs in genes under different selective constraints also show significant differences in functional consequences. We observed that chemical distance (Grantham 1974), change in amino acid hydrophobicity index (|*ΔH*|) (Argos et al. 1982), and BLOSUM62 (Henikoff S and Henikoff JG 1992) score are consistently higher for SNPs located in genes with higher ω value (fig. 2*E* and supplementary figs. S3 and S4, Supplementary Material online). Higher *ω* value suggests a relaxed selective constraint on protein coding sequence evolution and that SNPs in genes with weaker selective constraints have larger functional changes. This suggests purifying selection on rare SNPs is a major factor shaping genetic variation in the human genome.

We also investigated the DAF of human exonic SNPs in different genomic regions. We observed that the cumulative distribution of DAF is significantly different for SNPs in different genome regions (fig. 5*A*). The DAF of SNPs in coding region is significantly lower than the DAF of SNPs in 5′- and 3′-UTR s (fig. 5*A*). Also, we observed lower DAF for SNPs in TIS (fig. 5*B*) and SS (fig, 5*C* and *D*) compared with non-TIS and non-SS regions, respectively. In addition, we found that the DAF of SNPs in functionally essential genes (supplementary fig. S5*A*, Supplementary Material online) and Mendelian genes (supplementary fig. S5*B*, Supplementary Material online) is significantly lower than that in nonessential genes and non-Mendelian genes, respectively. The regional difference in DAF confirms the role of purifying selection in human genome evolution.

The excessive number of evolutionarily young SNPs with a low DAF (bottom left of fig. 6*A*) is consistent with recent explosion of human SNPs, as well as with the recent demographic history of human population (Gravel et al. 2011; Fu et al. 2013). Younger SNPs have lower DAF, which may have substantial functional and structural consequences. We also showed that younger SNPs are more likely to be found in conserved regions (fig. 6*B* and supplementary figs. S6*A* and S7*A*, Supplementary Material online). These results are compatible with previous findings that recent human SNPs are more likely to be rare and deleterious (Gravel et al. 2011; Fu et al. 2013).

Finally, we want to draw attention to the distinction between slow-running and quick-running SNPs in the human genome (fig. 6*A*). For the quick-running SNPs, the derived allele may have very high selective advantage over the ancestral allele, and can be fixed very quickly in the population. The PhyloP score of quick-running SNPs is significantly less than zero, which means those quick-running SNPs experienced positive selection. Curiously, those SNPs are enriched in genes performing olfactory transduction function (supplementary fig. S8, Supplementary Material online), which are known to be under positive selection in human evolution (Young and Trask 2002). Several other quick-running SNPs are located in genes coding Zinc-finger proteins, such as ZNF33B and ZNF460. Previous studies have also shown zinc-finger proteins have experienced lineage-specific positive selection in humans (Schmidt and Durrett 2004). Interestingly, slow-running SNPs have similar conservation scores as quick-running SNPs, and pathway analysis suggests that both these categories of SNPs are enriched in olfactory related genes (supplementary fig. S8, Supplementary Material online). The reason for these similar selective constraints is unknown. The ancestral alleles of human SNPs were inferred by the comparison of human genome to six-way primate genome alignment (Paten et al. 2008), which may lead to incorrect ancestral allele definition of some human SNPs. The misdefinition of ancestral allele of human SNPs could partially explain the signal of positive selection observed in slow-running SNPs. Furthermore, our results suggest that positive selection and genetic hitchhiking (Fay and Wu 2000) may account for the quick fixation of some “fast-running” SNPs. However, other possible mechanisms, such as weak background selection or chance alone, could also explain fast-running for some human SNPs. Hence, more thorough analysis will be needed to explore the exact mechanisms for the fast-running and slow-running SNPs.

In conclusion, our systematic analysis of functional and structural consequences of human exonic SNPs suggested that purifying selection, rather than positive or balancing selection, shapes much of the genetic variation in human populations. Rare exonic SNPs with DAF less than 1%, which appeared recently in the human population, cause the largest functional and structural changes, whereas rare exonic SNPs with DAF larger than 99% have the smallest functional consequences. This will have important implications for understanding the molecular underpinnings of complex diseases.

## Supplementary Material

Supplementary table S1 and figures S1–S12 are available at *Genome Biology and Evolution* online (http://www.gbe.oxfordjournals.org/).

Supplementary Data
