# Supplementary material for: Functional and Structural Consequence of Rare Exonic Single Nucleotide Polymorphisms: One Story, Two Tales
Source: Genome Biol Evol. 2015 Oct 9;7(10):2929–40. doi: 10.1093/gbe/evv191 (PMC4684694; doi:10.1093/gbe/evv191)
Supplement: Supplementary Data [file supp_7_10_2929__index.html]

Functional and Structural Consequence of Rare Exonic Single Nucleotide Polymorphisms: One Story, Two Tales — Supplementary Data 

# Functional and Structural Consequence of Rare Exonic Single Nucleotide Polymorphisms: One Story, Two Tales

## Supplementary Data

files

- Supplementary Data - pdf file
