# Supplementary material for: Functional and Structural Consequence of Rare Exonic Single Nucleotide Polymorphisms: One Story, Two Tales
Source: Genome Biol Evol. 2015 Oct 9;7(10):2929–40. doi: 10.1093/gbe/evv191 (PMC4684694; doi:10.1093/gbe/evv191)
Supplement: Supplementary Data [file supp_evv191_Supplementary_Materials.pdf]

Table S1. Human codon optimality

Amino acid	Codon	Optimality	Adjusted *P*-value
I	ATT	0.9618	3.76E-02
I	ATC	1.1409	5.72E-16
I	ATA	0.7364	7.67E-34
L	CTT	0.9459	3.43E-03
L	CTC	1.0243	1.26E-01
L	CTA	0.9247	1.85E-03
L	CTG	1.0722	1.56E-09
L	TTA	0.7740	2.24E-27
L	TTG	0.9868	4.78E-01
V	GTT	0.9099	7.03E-06
V	GTC	1.0163	3.84E-01
V	GTA	0.9025	5.58E-05
V	GTG	1.0529	3.38E-04
F	TTT	0.9458	2.96E-03
F	TTC	1.0477	8.34E-03
C	TGT	0.9437	1.66E-02
C	TGC	1.0477	3.76E-02
A	GCT	1.0145	3.84E-01
A	GCC	1.0335	1.69E-02
A	GCA	0.9656	4.53E-02
A	GCG	0.9222	4.88E-04
G	GGT	1.0538	1.17E-02
G	GGC	1.0378	1.39E-02
G	GGA	0.9093	3.44E-08
G	GGG	1.0048	7.75E-01
P	CCT	1.0310	7.50E-02
P	CCC	1.0163	3.30E-01
P	CCA	0.9752	1.51E-01
P	CCG	0.9437	1.69E-02
T	ACT	0.9522	1.39E-02
T	ACC	1.0714	5.58E-05
T	ACA	0.9374	6.96E-04
T	ACG	1.0415	1.26E-01
S	TCT	1.0352	5.68E-02
S	TCC	1.0468	7.24E-03
S	TCA	0.9262	1.39E-04
S	TCG	0.9875	6.76E-01
S	AGT	0.9265	1.12E-04
S	AGC	1.0289	7.29E-02
Y	TAT	0.9383	3.78E-03

Amino acid	Codon	Optimality	Adjusted *P*-value
Y	TAC	1.0505	1.39E-02
Q	CAA	0.7861	8.57E-33
Q	CAG	1.0853	5.01E-09
N	AAT	0.8866	6.14E-11
N	AAC	1.1100	2.46E-09
H	CAT	0.9414	1.09E-02
H	CAC	1.0417	4.53E-02
E	GAA	0.8879	1.25E-18
E	GAG	1.0883	2.45E-12
D	GAT	0.9846	3.47E-01
D	GAC	1.0133	3.84E-01
K	AAA	0.8549	7.60E-26
K	AAG	1.1213	1.25E-17
R	CGT	1.1653	3.41E-07
R	CGC	1.0561	8.63E-03
R	CGA	1.0987	3.52E-04
R	CGG	1.1164	1.73E-08
R	AGA	0.8167	6.47E-24
R	AGG	0.9110	7.21E-06

Note – *P*-values were calculated by χ^2 test and adjusted by Benjamini-Hochberg procedure.

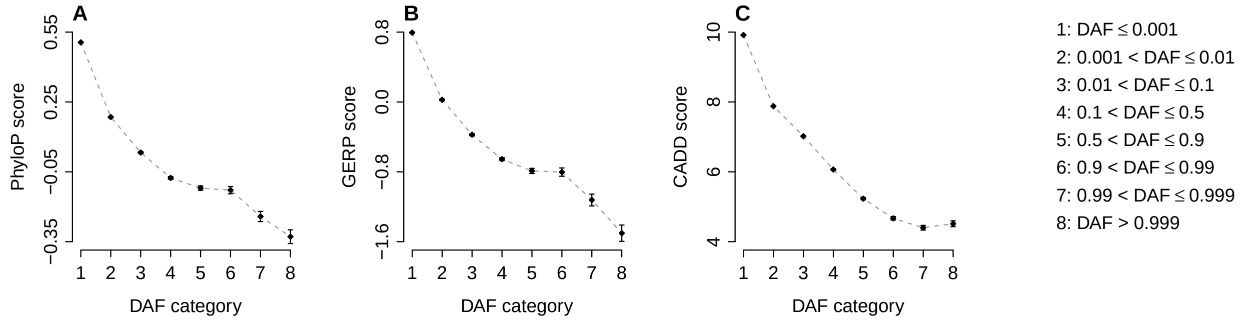

Figure S1. Relationship between DAF of exonic SNPs and corresponding conservation/pathogenicity score. (A) Relationship between DAF and PhyloP score; (B) Relationship between DAF and GERP score; (C) Relationship between DAF and CADD score. Each point stands for the mean of the corresponding category. Error bars indicate the standard error of the mean.

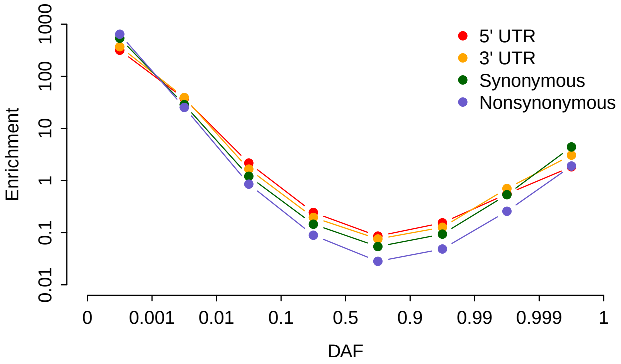

Figure S2. Enrichment of exonic SNPs at the both extremes of DAF. The enrichment value was calculated under the assumption that exonic SNPs are evenly distributed along DAF.

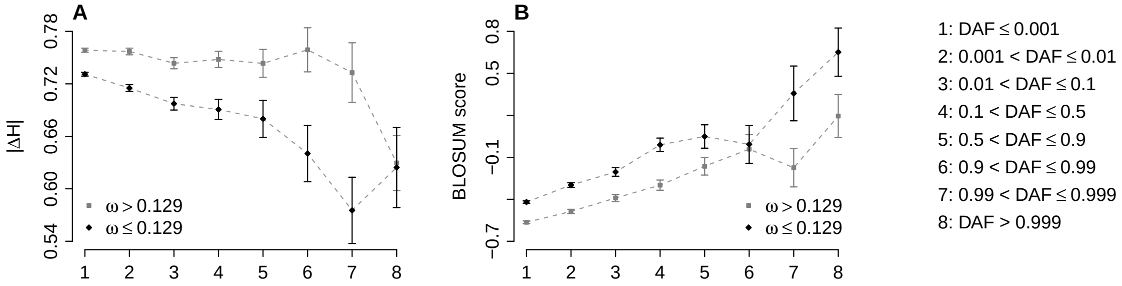

Figure S3. Comparison of the functional effect caused by nonsynonymous SNPs between the genes undergoing stronger and weaker purifying selection using mouse orthologs. The median of ω is used as the cutoff to stratify human genes as under stronger or weaker purifying selection. (A) Change in amino acid hydrophobicity index ($|\Delta H|$) as a function of DAF; (B) BLOSUM score between the altered amino acids as a function of DAF. Each point stands for the mean of the corresponding category. Error bars indicate the standard error of the mean.

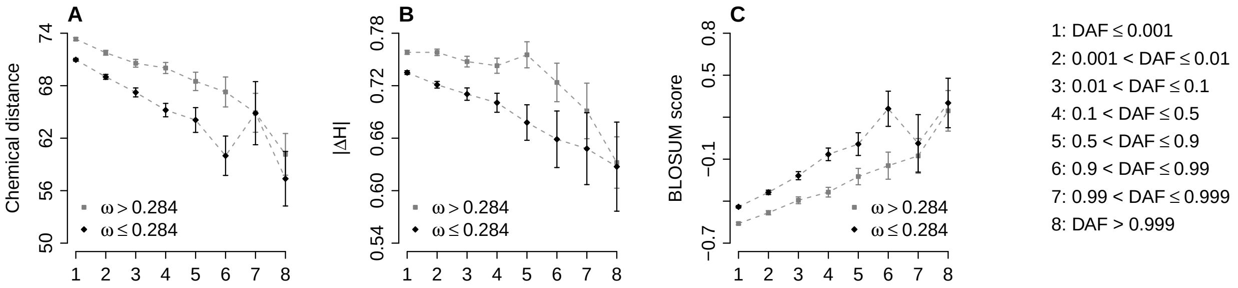

Figure S4. Comparison of the functional effect caused by nonsynonymous SNPs between the genes undergoing stronger and weaker purifying selection using chimp orthologs. The median of ω is used as the cutoff to stratify human genes as under stronger or weaker purifying selection. (A) Amino acid chemical distance caused by nonsynonymous SNPs as a function of DAF; (B) Change in amino acid hydrophobicity index ($|\Delta H|$) as a function of DAF; (C) BLOSUM score between the altered amino acids as a function of DAF. Each point stands for the mean of the corresponding category. Error bars indicate the standard error of the mean.

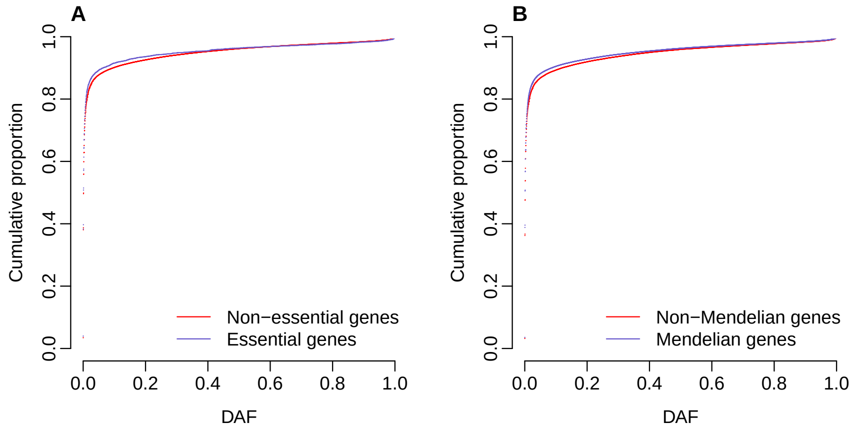

Figure S5. Cumulative distribution of DAF. (A) Comparison of DAF between SNPs located in essential and non-essential genes; (B) Comparison of DAF between SNPs located in Mendelian and non-mendelian genes.

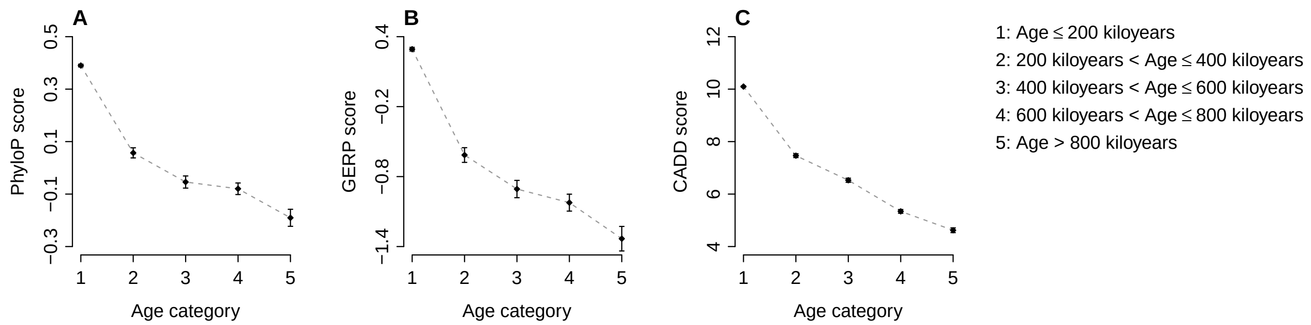

Figure S6. Relationship between SNP age in European American and corresponding conservation/pathogenicity score. (A) Relationship between age and PhyloP score; (B) Relationship between age and GERP score; (C) Relationship between age and CADD score. Each point stands for the mean of the corresponding category. Error bars indicate the standard error of the mean.

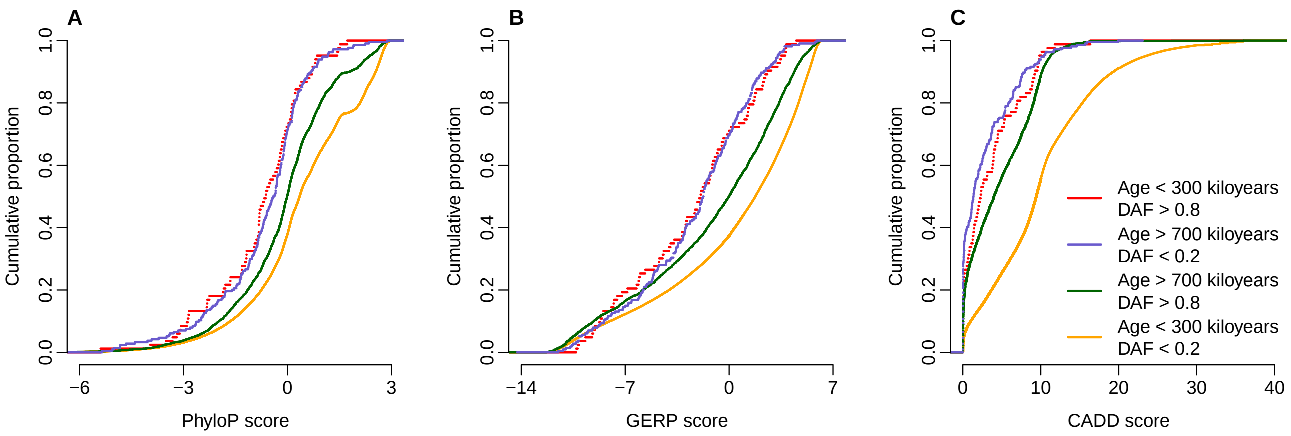

Figure S7. Cumulative distribution of conservation/pathogenicity score of the SNPs in the four corners of Figure 6A. (A) Cumulative distribution of PhyloP score; (B) Cumulative distribution of GERP score; (C) Cumulative distribution of CADD score.

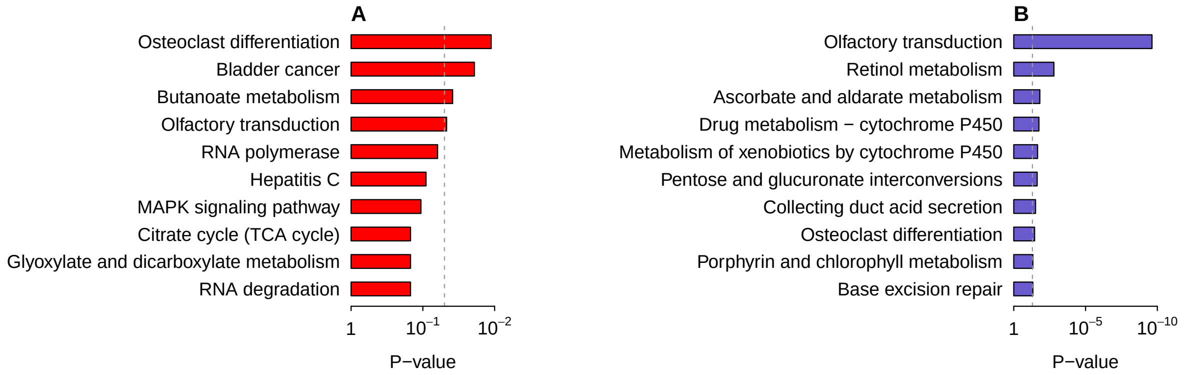

Figure S8. KEGG pathways associated with the "quick-running" and "slow-running" SNPs. (A) Pathways associated with the "quick-running" snps; (B) Pathways associated with the "slow-running" snps. The grey dash line indicates the α level of 0.05.

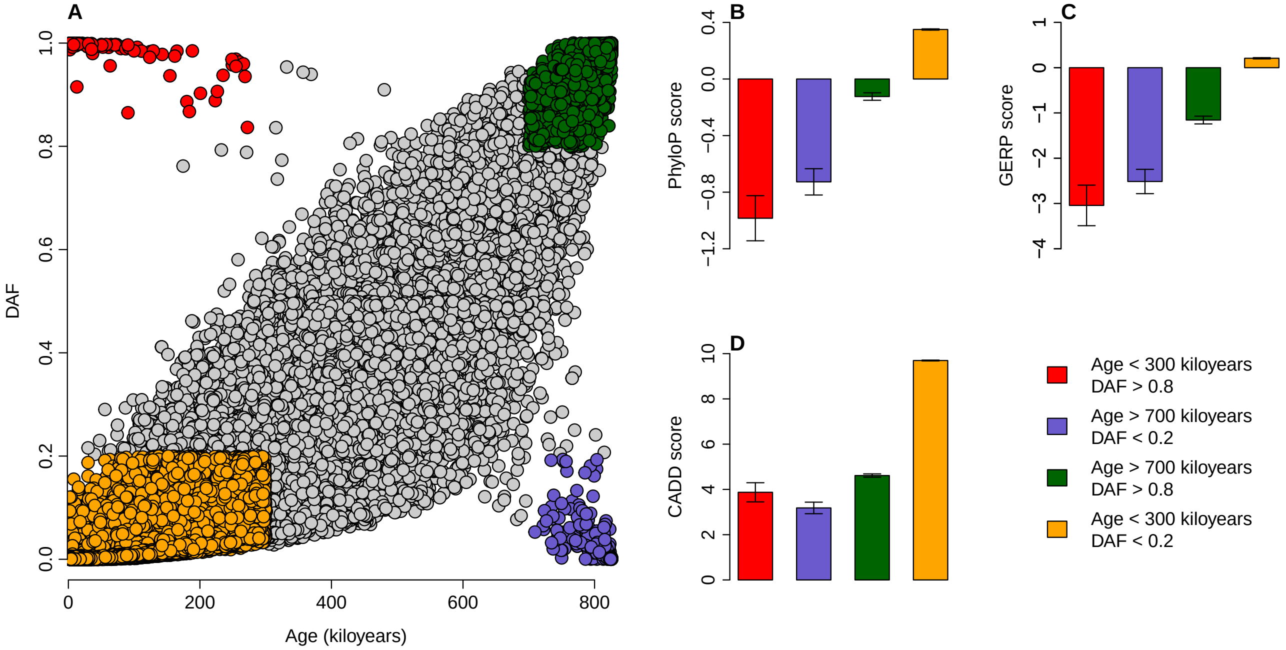

Figure S9. Relationship between SNP age in African American and DAF. (A) DAF increases with SNP age. Outliers are aggregated at the top left (red points with age $<$ 300 kiloyears and DAF $>$ 0.8) and bottom right (blue points with age $>$ 700 kiloyears and DAF $<$ 0.2) corners. The SNPs located in the top right (green points with age $>$ 700 kiloyears and DAF $>$ 0.8) and bottom left corners (orange points with age $<$ 300 kiloyears and DAF $<$ 0.2) are highlighted. (B) Comparison of PhyloP score among the SNPs located in the four corners in panel A. (C) Comparison of GERP score among the SNPs located in the four corners in panel A. (D) Comparison of CADD score among the SNPs located in the four corners in panel A.

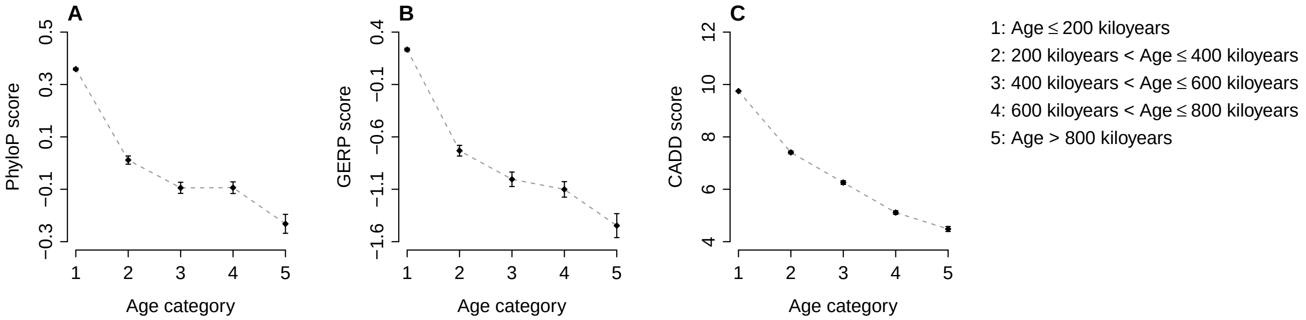

Figure S10. Relationship between SNP age in African American and corresponding conservation/pathogenicity score. (A) Relationship between age and PhyloP score; (B) Relationship between age and GERP score; (C) Relationship between age and CADD score. Each point stands for the mean of the corresponding category. Error bars indicate the standard error of the mean.

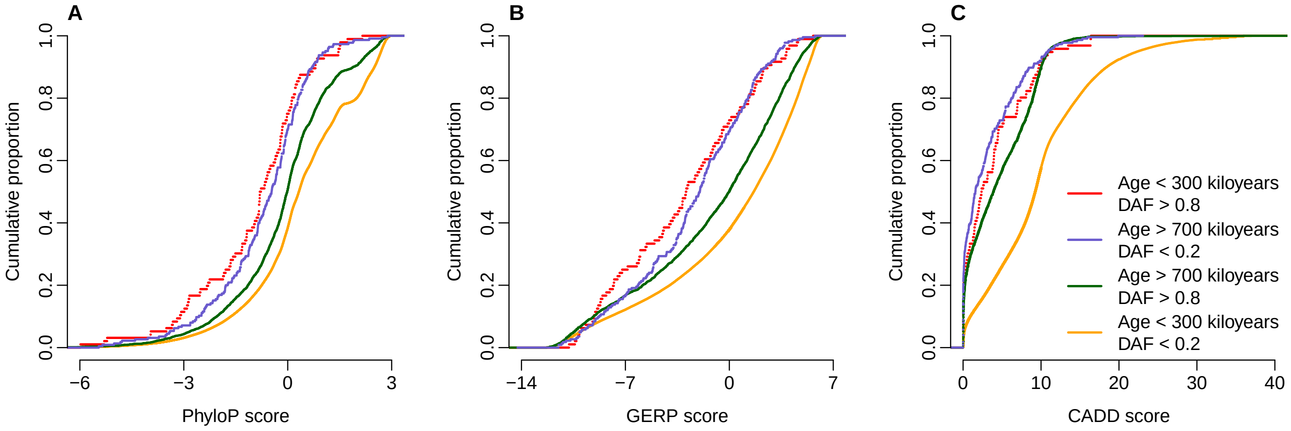

Figure S11. Cumulative distribution of conservation/pathogenicity score of the SNPs in the four corners of Figure S9. (A) Cumulative distribution of PhyloP score; (B) Cumulative distribution of GERP score; (C) Cumulative distribution of CADD score.

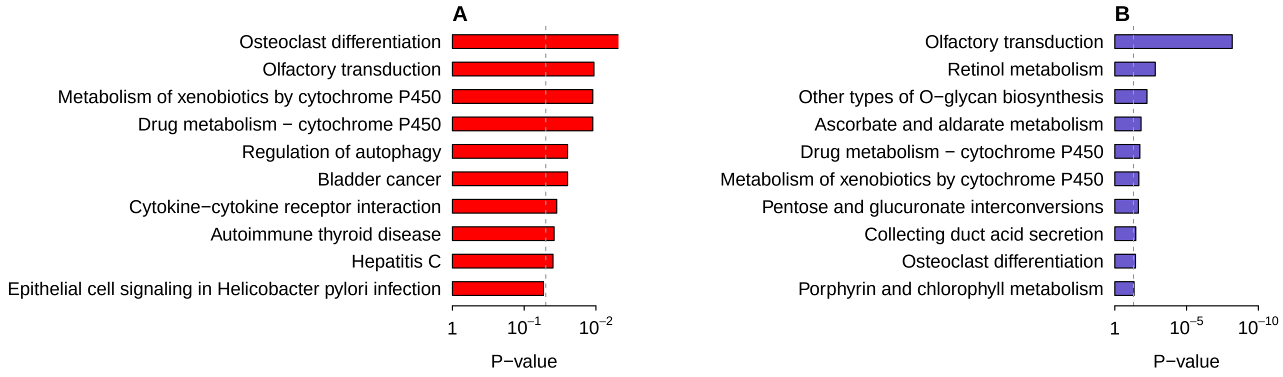

Figure S12. KEGG pathways associated with the "quick-running" and "slow-running" SNPs in African American. (A) Pathways associated with the "quick-running" snps; (B) Pathways associated with the "slow-running" snps. The grey dash line indicates the α level of 0.05.
